# Smartphone Technology to Facilitate Remote Postural Balance Assessment in Acute Concussion Management: Pilot Study

**DOI:** 10.3390/s24216870

**Published:** 2024-10-26

**Authors:** Oren Tirosh, Jaymee Klonis, Megan Hamilton, John Olver, Nilmini Wickramasinghe, Dean Mckenzie, Doa El-Ansary, Gavin Williams

**Affiliations:** 1 School of Health and Biomedical Sciences, The Royal Melbourne Institute of Technology University, 225-245 Plenty Rd, Bundoora, VIC 3082, Australia; doa.el-ansary@rmit.edu.au; 2 Rehabilitation Sciences, Shanghai University of Medicine and Health Sciences, Shanghai 200235, China; 3 Epworth Concussion Clinic, Epworth Hospital, Richmond, VIC 3121, Australia; jaymee.klonis@epworth.org.au (J.K.); megan.hamilton@epworth.org.au (M.H.); john.olver@epworth.org.au (J.O.); dean.mckenzie@monash.edu (D.M.); gavin.williams@epworth.org.au (G.W.); 4 School of Computing, Engineering and Mathematical Sciences, La Trobe University, Bundoora, VIC 3086, Australia; nilmini.work@gmail.com; 5 Epworth Monash Rehabilitation Medicine Unit, University of Melbourne, Melbourne, VIC 3000, Australia; 6 Department of Physiotherapy, University of Melbourne, Grattan Street, Parkville, Melbourne, VIC 3010, Australia

**Keywords:** concussion, balance, smartphone, remote assessment, return to sport

## Abstract

Impaired balance is a key symptom following acute concussion. Unfortunately, the recommended clinical balance assessment lacks sensitivity and discriminative ability, relying on the experience of the clinician for interpretation. The aim of this pilot study is to explore smartphone technology to remotely assess balance impairment in people with acute concussion. A smartphone app was developed to allow the clinician to connect remotely using their personal web browser to the participant’s smartphone and collect motion data while instructing the participant to perform the following balance tests: standing on firm and foam surface with eyes opened and closed (FIRMEO, FIRMEC, FOAMEO, and FOAMEC). Outcome measures were processed from the raw acceleration to calculate the average acceleration magnitude from the mean and the root-mean square, with greater values indicating more sway. Eleven healthy controls (HCs) and 11 people with concussion (CON) participated. In all sway measurements, the CON group had significantly (*p* < 0.05) greater values when standing on a firm surface. In the FOAMEC condition, the CON group had significantly (*p* < 0.05) greater sway measures only in the AP direction, while significantly greater sway in all directions were found in the CON group in the FOAMEO condition. This study shows that remote balance assessment using a smartphone can discriminate between healthy controls and people with acute concussion. This technology could play an important role in concussion management to assist with determining recovery from concussions and the optimal timing for return to sport.

## 1. Introduction

Concussion is a subset of mild traumatic brain injury (TBI) and sport-related concussion and accounts for 20% of all Australian TBIs [1]. Impaired balance is one of the most common symptoms [1], and balance assessment provides an indirect means of identifying concussion-related neurophysiological abnormality. The ability to balance and steadiness when walking with divided attention (cognitive) have been identified as an appropriate measures of assessing the physical sequelae of concussion and recovery. They are recommended as key features of a clinical concussion evaluation [2,3,4] and readiness to resume competitive activity [5]. 

Unfortunately, current clinical practice balance assessments lack sensitivity and discriminative power, relying on the experience of the clinician for scoring and interpretation [6,7]. ‘Gold standard’ force plate (~AUD 15k) testing is expensive and not practical in a clinical setting. The use of affordable accelerometer devices was proposed as a valid and reliable option to measure balance in individuals with concussion [8,9]. They have been shown to be sensitive and to discriminate balance tasks of varying degrees of difficulty in healthy adults, and are thus suitable for assessing TBI [10]. Unfortunately, the use of accelerometers requires assistance from personnel to operate, analyze, and interpret the data.

With the progress of technology, smartphones can serve as an alternative solution to the use of the aforementioned technology. Smartphones are ubiquitous, portable, and affordable, and have been reported to assess postural balance in various clinical populations. Smartphones also offer the potential to perform remote assessments in home environments and are equipped with inertial sensors [11]. Some studies have reported the use of smartphones in assessing dynamic gait balance among people with concussion [12,13]. To our knowledge, the use of smartphones to assess static postural balance in people with concussions has not been reported. Moreover, there is no evidence in the literature on performing postural balance assessment in a remote approach, involving the clinician connecting to the patient’s smartphone at home from their clinic web browser and performing the assessment in real-time, to enable assessment of those living remotely.

A nexus of smartphones and a web-based repository system, TelePhysio (Version 1.0), was developed and built to provide solutions for a much-needed digital health platform for objective remote postural balance assessments. TelePhysio uses smartphone inertial sensors to measure the linear acceleration (accelerometer) and angular velocity (gyroscope) that is captured during postural balance tasks and then can be used to quantify the level of performance by well-defined signal processing methods that are highly sensitive to any postural sway changes [14,15,16,17]. With TelePhysio, the clinician connects remotely using their clinic web browser to the participant’s smartphone and collects motion data while instructing the participant to perform a balance task. User usability and acceptance of TelePhysio with its validity and reliability were initially studied in healthy controls prior to evaluation on people with hip and knee musculoskeletal injuries [18,19,20,21].

The purpose of this pilot study was to assess if balance measurements from the TelePhysio smartphone technology were sensitive to differentiation between healthy controls and people with concussion.

This is the first study to use a telehealth smartphone application to collect and share balance data via linking database infrastructure to enhance the management of concussion. This technology is new and can change current clinical practice for balance assessment administration from a subjective, nondigital, and face-to-face system to an advanced, objective, digital, and remote data collection technology that can encompass all communities and provide a research platform to advance evidence-based solutions. This innovative solution provides tele-assessment in concussion management, which telehealth is currently lacking around the world. 

## 2. Materials and Methods

### 2.1. Participants

A total of 11 healthy adults (HC, 2 males, 9 females, 36.6 ± 11.4, 5 years, range of 26–66 years) and 11 participants with concussion (CON, 6 males, 5 females, 36.8 ± 13.5 years, range of 15–64 years) were recruited for the study. All participants were English-speaking. The inclusion criteria for CON participants were as follows: (a) ≥14 years of age, (b) have sustained a concussion injury (as diagnosed by the referring doctor), (c) have proficient English language skills (i.e., do not require an interpreter), and (d) have a mobile smartphone. The exclusion criteria included concurrent medical condition that may affect the ability to balance or walk. The HC participants in this study (a) had no previous concussion or brain injury, (b) had no balance or mobility limitations, and (c) met the matching criteria. Ethical approval was granted by the Royal Melbourne Hospital Human Research Ethics Committee (ref: HREC/82346/MH-2021). All participants provided signed written informed consent prior to testing.

### 2.2. Instrumentation

**Figure 1:**
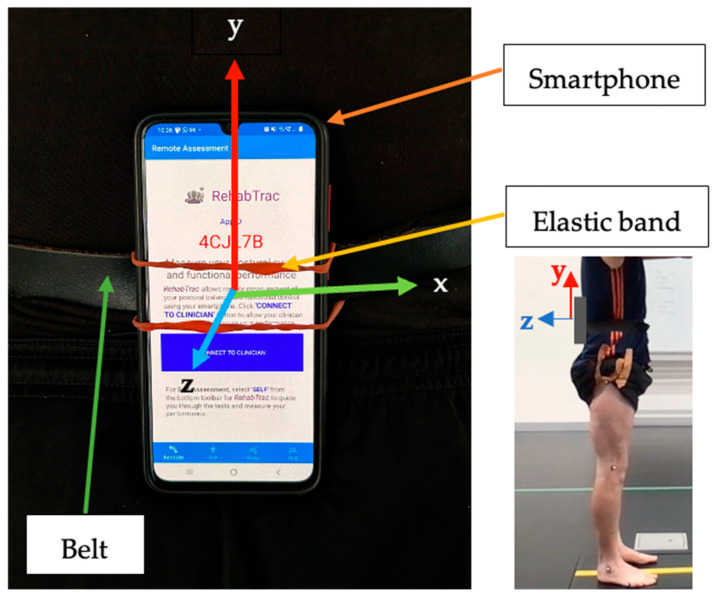
An illustration of smartphone attachment using an elastic band attached to a belt around the waist.

All balance and walking assessments were measured using the smartphone TelePhysio app, controlled by the clinician from their web browser. TelePhysio is our original tele-assessment platform that uses smartphone motion sensors to allow clinicians to measure and assess the real-time functional performance of their patients in a remote and objective way [19,20,21]. For the CON group, the assessment was performed, on average, 22 days following a concussion. At the beginning of the assessment, the examiner remotely connected using their web browser to the participant’s TelePhysio app that was installed on the participant’s smartphone. The smartphone was attached using an elastic band to a belt that was strapped around the participant’s waist, positioned at their lower back with the smartphone screen facing away from the body in a portrait layout, with the top side facing upwards (see Figure 1). All participants used their personal smartphones; thus, the smartphones were not of any specific brand model.

**Figure 2:**
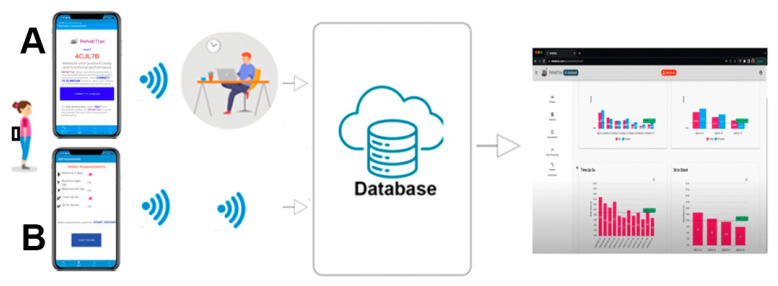
An overview of the app, using the real-time (**A**) and offline (**B**) modules from testing to analysis and interpretation using the Cloud database. The smartphone is attached to the lower back.

A software bundle of the Cloud repository interface, smartphone motion sensors, and the app was developed to provide solutions for much-needed digital health data infrastructure for objective remote balance assessments. Figure 2 illustrates the process of remote balance assessment using the Cloud and the app. The technology platform uses smartphone motion sensors to measure linear accelerations (accelerometer) and angular velocities (gyroscope) captured during a balance task, and then uploads and stores these data in the Cloud to quantify performance levels by well-defined signal processing methods that are highly sensitive to postural sway changes [22]. Both the clinician and patient can then log in using a web browser to view the results. The app provides two motion data capture modules, including (1) real-time and (2) offline. With the real-time module, the clinician connects remotely using their web browser to the participant’s smartphone that is placed at the lower back and collects motion data, while instructing the participant to perform the balance task. With the offline module, the participant self-administers their assessment program without the need for clinician interaction. By selecting from the list of balance tasks, the app audio guides the user through the list of tasks while measuring their performance and automatically transfers the sensor data to the Cloud to further analyze and instantly feed back the level of performance and balance impairment. This way, the user receives immediate feedback and becomes empowered and enabled to take more control of their recovery process while the clinician can regularly evaluate the progress of concussion.

### 2.3. Protocol

Participants attended a 30 min testing session. The participant turned on the TelePhysio app and attached the smartphone using an elastic band to a belt that was strapped around the waist, such that the smartphone was positioned at the lower back. The clinician then connected remotely to the TelePhysio app from their clinic web browser and collected motion data whilst the participant performed the balance and walking tasks. Once a specific task was completed, the clinician uploaded the smartphone sensor’s data to the web-based Cloud application for later processing and analysis. During the second session, voice communication between the examiner and participant was conducted via Zoom (Zoom Video Communications, Inc., San Jose, CA, USA).

Participants were asked to perform the following postural balance and walking tasks:

(a) Modified CTSIB: This test involves four balance tests, including (1) standing on a firm surface with eyes open (FIRMEO), (2) standing on a firm surface with eyes closed (FIRMEC), (3) standing on a foam surface with eyes open (FOAMEO), and (4) standing on a foam surface with eyes closed (FOAMEC). Each test was performed for up to 30 s with the participant’s feet together and their hands on their hips. The test was terminated if a participant moved their hands or feet. Each participant was given 3 attempts to reach 30 s. If they were unable to make 30 s, the average of the 3 attempts was recorded.
(b) Heel–toe (tandem) walking (TANDEM): The participants were instructed to walk forward along a 3 m long piece of sports tape, 38 mm wide, with an alternate heel-to-toe gait, where the heel and toe were approximated on each step. Once they crossed beyond the end of the 3 m piece of tape, they were instructed to turn and return to the original starting point with the same gait pattern and to complete the trial as fast as possible without stepping off the line, having a separation between the heel and toe, or touching the test administrator. The time taken to perform this test was recorded. Participants had 1 familiarization trial before 1 recorded trial.
(c) Heel–toe (tandem) walking with cognitive interference (TANDEMCOG): This test was the same as the heel–toe (tandem) walking test above, but with an additional cognitive interference task. Whilst walking heel–toe, participants were asked to state the months of the year in reverse order. The time taken to perform this test was recorded. Participants had 1 familiarization trial before 1 recorded trial.

### 2.4. Data Analysis

All accelerometer and gyroscope signals from the smartphone were processed using R-Statistics version 4.2.1 [23]. For the balance tests, the outcome measurements from TelePhysio included the following: (1) the average acceleration magnitude from the mean (aam) for the medio-lateral (aamml) and anterior–posterior (aamap) directions and (2) the root-mean-square acceleration for ML (rmsml) and AP (rmsap). For the tandem walking tests, the outcome measures included the following: (1) aamml and aamap, (2) rmsml and rmsap, (3) the sample entropy (saen) of the angular velocity, with lower values indicating that the movement is more regular, repetitive, and predictable, (4) the time to turn (tturn), and (5) the total time for completion from the start to begging of the turn (ttotal). All acceleration measurements were calculated for the medio-lateral (ML) and anterior–posterior (AP) directions. The acceleration signal processing and calculation of the aam and rms measurements were performed as described previously [22]. Briefly, the gravity component was eliminated by subtracting the acceleration signal mean, followed by a zero-phase Butterworth high-pass filter at 0.3 Hz, and then a third-order Savitzky–Golay smoothing filter with frames of 41 points. Sample entropy was calculated only for the TANDEM and TANDEMCOG walking assessments to quantify the amount of regularity and unpredictability of fluctuations, as described earlier for dynamic tasks for people with concussion [24].

**Figure 3:**
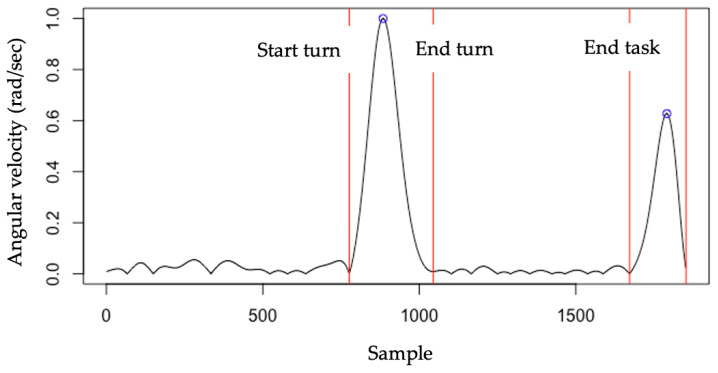
The identification of turning during the tandem walking trials using angular velocity. The angular velocity was processed by first filtering with a Butterworth lowpass filter (10 Hz, 4th order, and zerolag), followed by rectifying the signal and normalizing it to its maximum peak value. The first red vertical line represents the beginning of the turn and the end of walking. The second red vertical line represents the end of the turn and the start of walking. The third vertical line represents the start of a turn and the end of walking.

To identify the beginning and end of turning and the time to completion during the tandem walking, the x-axis angular velocity from the smartphone gyroscope signal was used (see Figure 3). Similar to Beyea et al.’s [25] study, the angular velocity was processed by first filtering with a Butterworth low-pass filter (10 Hz, 4th order, and zero-lag), followed by rectifying the signal and normalizing it to its maximum peak value. Figure 3 shows the identification of the turning in a sample tandem walking trial. The first angular velocity peak represents the turning and the second peak represents the second turn end of the trial. The turning time was calculated from the start to the end of the first turning.

### 2.5. Statistical Analysis

To explore significant group and testing task effects and interaction, a two-way ANOVA was performed for the postural balance trials, group (CON, HC) X test (FIRMEO, FIRMEC, FOAMEO, FOAMEC), and for the tandem walking trials, group (CON, HC) X test (TANDEM, TANDEMCOG). A Bonferroni post hoc analysis was performed to identify differences between the groups at the 4 balance tasks and the 2 tandem walking tasks. All analyses were performed using the free software Statistical Package R version 4.2.1 (https://www.r-project.org/ (accessed on 8 July 2022)) with the significance level set at 0.05.

## 3. Results

Analysis showed a significant balance test effect (*p* < 0.05) for all measurements, with the lowest sway measures in the FIRMEO condition followed by FIRMEC, FOAMEO, and FOAMEC conditions. Similarly, a significant group effect (*p* < 0.05) with no significant group X Test interaction was found in all measurements, indicating greater sway in the CON group. Table 1 presents balance outcomes for the HC and CON groups for all balance testing conditions. In all sway measurements, the CON group had significantly (*p* < 0.05) greater values while standing on a firm surface (FIRMEO and FIRMEC). When standing on foam with the eyes closed, the CON group had significantly (*p* < 0.05) greater sway measures only in the AP direction, while significantly greater sway in all directions was found in the CON group when standing on foam with the eyes opened.

Table 2 presents the outcome measures during the two tandem walking conditions. Significantly (*p* < 0.05) lower values for all measurements, except for the time to turn, were found in the non-cognitive tandem walking group (TANDEM) compared to the cognitive task tandem walking group (TANDEMCOG). Tandem walking did not show a significant effect between groups, as seen with HC compared with CON (15.5 ± 7.4 s and 11.3 ± 3.4 s, respectively) and non-cognitive tests (10.9 ± 4.9 s and 8.2 ± 2.8 s).

## 4. Discussion

This study found significantly greater sway measures in both medio-lateral and anterior–posterior directions in people with concussions in all four balance tests; standing on firm and foam surfaces with the eyes opened and closed. The results of this study show that smartphone technology can measure balance remotely and discriminate balance performance between people with concussion and healthy controls.

To identify balance impairments, clinicians constrain the sensory system by asking patients to close their eyes (vision constrain) and/or stand on a foam surface (proprioceptor constrain). The smartphone technology in this study was able to discriminate between the level of standing balance difficulties such as standing on a firm surface with the eyes open and closed, and on a foam surface with the eyes open and closed. Therefore, this technology has the potential to be used in other populations to identify the effect of sensory constraints on balance.

The Balance Error Scoring System (BESS) is the clinical gold standard for assessing the impact of concussion on balance. However, the BESS test lacks sensitivity and discriminative power, and the ability to detect balance problems after the third day of recovery [26,27]. This is problematic and impairs clinician judgment, as research has found that for most injured athletes, symptoms continue at least during the first 2 weeks following injury [26,28]. The core issue of the BESS is its subjective nature. More specifically, the known that the limitations of the BESS assessment include the following: (a) subjective scoring that depends on administrator expertise, (b) poor to moderate reliability and validity [27], (c) poor sensitivity (34–64%) that can only detect balance deficits where large differences exist, thus not being suitable when differences are more subtle [29,30], (d) an inability ti identify the specific sway directions deficit, such as sway to the medio-lateral (ML) or to the anterior–posterior (AP) directions, and (e) difficulty to perform remotely.

Our proposed technology could be a superior option to the BESS. The technology utilizes the inertial sensors embedded in the smartphone, placed at the lower back, to measure postural sway during the balance task. The use of inertial sensor devices placed at the lower back to measure balance was shown to produce objective, reliable, and highly validated measurements [8,31], and to be sensitive to small postural sway changes in both ML and AP directions, with better discrimination between those with and without post-concussive symptoms than the standard clinical BESS error count [31]. Previously, we found that our smartphone technology was reliable in measuring postural sway during double and single-leg squats in adults with Femoroacetabular Impingement and healthy controls [19]. Here, we extended the use of the smartphone app’s capabilities to measure balance in people with concussions and to allow remote assessment functionality. Today, governments have recognized that the health care system needs to have equity of access. This means that health care is easily accessible when needed and equitably distributed amongst the population. The TelePhysio app offers two advantages; not only is it potentially superior to the BESS, but it can be used remotely to provide objective data collection to allow clinicians to rely on evidence-based data to improve diagnoses and monitor balance changes post-concussion, while not being limited to only those that can geographically attend a concussion expert and/or can afford to. The proposed smartphone platform provides a solution to equity of access.

The tandem test with and without a cognitive task is another assessment that is recommended to assess gait function in people with concussion [32], and the overall time for completion is measured. Our results are comparable to previous reports on tandem gait in healthy controls and people with concussion, suggesting that smartphone technology is capable of measuring tandem gait remotely. In addition, this study found a significantly greater completion time in the cognitive tandem gait task compared to the non-cognitive task, showing the ability of the technology to differentiate between tasks at different levels of challenge.

It is reported that tandem gait performance is significantly impaired acutely following concussion [32]. In this pilot study, people with concussion had a greater, but not significant, completion time compared to healthy controls in cognitive tandem walking. Our finding of significant group differences in balance assessments but not in tandem test results may be surprising, and it can be argued that performing a balance test is sufficient to assess people with concussions. Murray et al. [33] reported that athletes with concussions took longer to complete tandem tests compared to healthy controls. Conversely, like our results, Santo et al. [34] reported that there was no difference in the best time between those with and without a history of concussion. Discrepancies between Murry et al.’s study and this study may be due to the time of testing following concussion (2 days and 22 days for Murry and this study, respectively), and the sample size (30 and 11, respectively). It may be that after 22 days, the concussion symptoms of this study’s participants reduced such that they had less effect on balance while walking. 

Some caution with the interpretation of the tandem walking results, however, is required, as it was previously recommended that the tandem gait test may lead to a high false-positive rate in healthy, physically active adults [34]. In addition, the authors reported high variability with turning time measurement, which may suggest the that turning time is not a good indicator of performance. The high variability in turning times may be related to our non-significant difference findings in the turning time between the groups. In addition, the small sample size in this study is a limitation that may have affected the non-significant overall results in tandem gait assessments, indicating the need for future investigation with a larger sample size.

Future studies should incorporate balance assessments throughout several weeks following concussion onset to identify if concussion persists and assist in return-to-play decisions. Smartphone technology has the potential to facilitate the use of easy-to-use remote and objective tools to follow people with a concussion, and future studies may explore longitudinal differences between the BESS and the TelePhysio balance assessment.

## 5. Conclusions

The use of smartphone technology to measure balance is sensitive to distinguishing between healthy controls and people with acute concussion. Remote tele-assessment using a smartphone application to measure postural sway can significantly discriminate between levels of standing difficulties, including standing on a firm surface with the eyes open, on a firm surface with the eyes closed, on a foam surface with the eyes open, and on a foam surface with the eyes closed. The use of a smartphone to remotely assess balance performance may be an appropriate and potentially effective alternative to face-to-face assessment.

## Figures and Tables

**Table 1:** Balance measures using a smartphone of healthy controls (HC) and people with concussion (CON) when standing for 30 s on a firm surface with the eyes open (FIRMEO), on a firm surface with the eyes closed (FIRMEC), on a foam surface with the eyes open (FOAMEO), and on a foam surface with the eyes closed (FOAMEC).

Measurement	ANOVA	Test	HC	CON	*p*-Value
	Group *F = 6.368 p* < 0.01 Test *F = 25.552* *p* < 0.001	FIRMEO	0.015 (0.030)	0.025 (0.012)	**0.013**
aamml		FIRMEC	0.022 (0.006)	0.046 (0.030)	**0.019**
		FOAMEO	0.022 (0.009)	0.046 (0.031)	**0.023**
		FOAMEC	0.060 (0.019)	0.100 (0.073)	0.094
	Group *F = 5.615 p* < 0.01 Test *F = 20.645* *p* < 0.001	FIRMEO	0.016 (0.004)	0.031 (0.016)	**0.005**
aamap		FIRMEC	0.027 (0.011)	0.057 (0.046)	**0.046**
		FOAMEO	0.019 (0.006)	0.044 (0.037)	**0.045**
		FOAMEC	0.049 (0.014)	0.106 (0.087)	**0.042**
	Group *F = 7.058 p* < 0.01 Test F *= 23.757* *p* < 0.001	FIRMEO	0.019 (0.004)	0.035 (0.019)	**0.012**
rmsml		FIRMEC	0.028 (0.008)	0.063 (0.044)	**0.017**
		FOAMEO	0.029 (0.011)	0.063 (0.046)	**0.026**
		FOAMEC	0.076 (0.023)	0.130 (0.093)	0.073
	Group *F = 5.690 p* < 0.01 Test *F = 18.314* *p* < 0.001	FIRMEO	0.020 (0.005)	0.043 (0.024)	**0.007**
rmsap		FIRMEC	0.034 (0.013)	0.076 (0.060)	**0.031**
		FOAMEO	0.025 (0.008)	0.059 (0.054)	0.054
		FOAMEC	0.063 (0.017)	0.141 (0.120)	**0.044**

*F* is the F-Value of the Analysis of Variance statistics. Significant *p* values (*p* < 0.05) are in bold.

**Table 2:** Outcome measures using a smartphone of healthy controls (HC) and people with concussions (CON) when performing tandem walking with (TANDEMCOG) and without (TANDEM) a cognitive task.

Measurement	Test	HC	CON	*p*-Value
	TANDEM	0.325 (0.078)	0.323 (0.113)	0.952
aamml	TANDEMCOG	0.281 (0.072)	0.280 (0.121)	0.972
	TANDEM	0.415 (0.114)	0.472 (0.139)	0.310
aamap	TANDEMCOG	0.361 (0.083)	0.376 (0.097)	0.691
	TANDEM	1.020 (0.008)	1.022 (0.015)	0.661
rmsml	TANDEMCOG	1.016 (0.008)	1.019 (0.021)	0.629
	TANDEM	1.031 (0.015)	1.040 (0.021)	0.244
rmsap	TANDEMCOG	1.025 (0.009)	1.027 (0.012)	0.582
	TANDEM	0.364 (0.213)	0.331 (0.192)	0.701
saen	TANDEMCOG	0.265 (0.138)	0.210 (0.100)	0.302
	TANDEM	8.214 (2.841)	10.897 (4.853)	0.129
ttotal	TANDEMCOG	11.291 (3.399)	15.543 (7.39)	0.098
	TANDEM	3.701 (1.544)	2.981 (0.514)	0.158
tturn	TANDEMCOG	3.764 (1.524)	3.342 (7.337)	0.473

## Data Availability

The raw data supporting the conclusions of this article will be made available by the authors on request and Supplementary Materials.

## Supplementary Materials

The following supporting information can be downloaded at: https://www.mdpi.com/article/10.3390/s24216870/s1.
